# Advances in the application of human amniotic membrane for tissue repair

**DOI:** 10.1186/s13619-026-00290-0

**Published:** 2026-05-09

**Authors:** Jiatong Han, Xinyue Yang, Arooj Khabbir, Chun Hu, Yidan Gao, Xinran Liu, Renping Liu, Yuyang Zhang, Yang Lei

**Affiliations:** 1 Queen Mary School, Jiangxi Medical College, Nanchang University, No. 1299, Xuefu Road, Honggutan District, Nanchang, China; 2 International Exchange College, Jiangxi Medical College, Nanchang University, Nanchang, China; 3 Department of Cardiovascular Medicine, The First Affiliated Hospital, School of Basic Medical Sciences and Institute of Biomedical Innovation, Jiangxi Medical College, Nanchang University, No. 1299, Xuefu Road, Honggutan District, Nanchang, China; 4 School of Basic Medical Sciences, Jiangxi Medical College, Nanchang University, No. 1299, Xuefu Road, Honggutan District, Nanchang, China; 5 Jiangxi Hypertension Research Institute, Nanchang, China; 6 Academician Workstation of Cardiovascular Innovative Materials, Nanchang, China

**Keywords:** Amniotic membrane, Orthopaedics, Obstetrics and gynaecology, Dermatology, Biomaterials

## Abstract

Human amniotic membrane (hAM) is a widely used biomaterial with longstanding utility in ophthalmology and emerging therapeutic promise across orthopaedics, obstetrics and gynaecology, and dermatology. Clinically available biological tissue repair materials primarily encompass autologous, allogeneic, and xenogeneic tissues. However, autologous materials are limited by availability, while allogeneic and xenogeneic tissues often present challenges related to immunocompatibility. As a commonly used allogeneic biomaterial, the hAM is regarded as a highly promising tissue repair material owing to its favourable immunological profile and exceptional tissue-regenerative properties. HAM is the innermost natural barrier of the placenta and possesses unique structural and biological characteristics that facilitate tissue repair and regeneration. This review summarizes recent advances and clinical applications of hAM in orthopaedics, obstetrics and gynaecology, and dermatology, specifically focusing on its roles in promoting tendon repair, alleviating osteoarthritis, repairing endometrial injury, treating diabetic foot ulcer, and enhancing burn wound healing. With the continued development of regenerative medicine, hAM is expected to play an increasingly important role in diverse tissue repair and regenerative medicine applications.

## Background

**Fig. 1:**
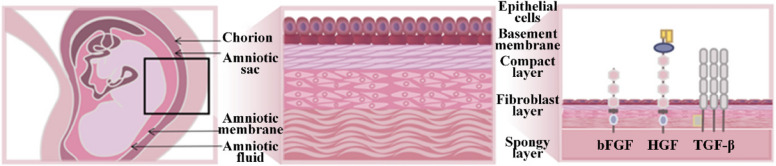
Schematic diagram of the structure of AM. bFGF, Basic fibroblast growth factor; HGF, hepatocyte growth factor; TGF‑β, transforming growth factor‑β. These growth factors are primarily secreted by epithelial cells and are mainly distributed in the basement membrane, epithelial and mesenchymal stromal cell layers (Arki et al. 2023)

As the innermost natural barrier of the placenta, the amniotic membrane (AM) has a thickness ranging from approximately 0.02 to 0.5 mm. Structurally, from superficial to deep, the AM consists of an epithelial layer, a basement membrane, and avascular stromal layers (Fig. 1) (Parmar et al. 2025). The extracellular matrix of the AM is predominantly composed of collagen and harbours mesenchymal stem cells that possess multilineage differentiation potential (Fanni et al. 2021; Litwiniuk and Grzela 2014). Moreover, as a natural allogeneic tissue, the AM exhibits low immunogenicity and a remarkable capacity for promoting tissue regeneration (Fenelon et al. 2021). Collectively, these unique biological and structural characteristics have established the AM as a successful therapeutic biomaterial in ophthalmology, while also providing a strong rationale for its expanding application in diverse clinical fields, including orthopaedics, obstetrics and gynaecology, and dermatology.

**Fig. 2:**
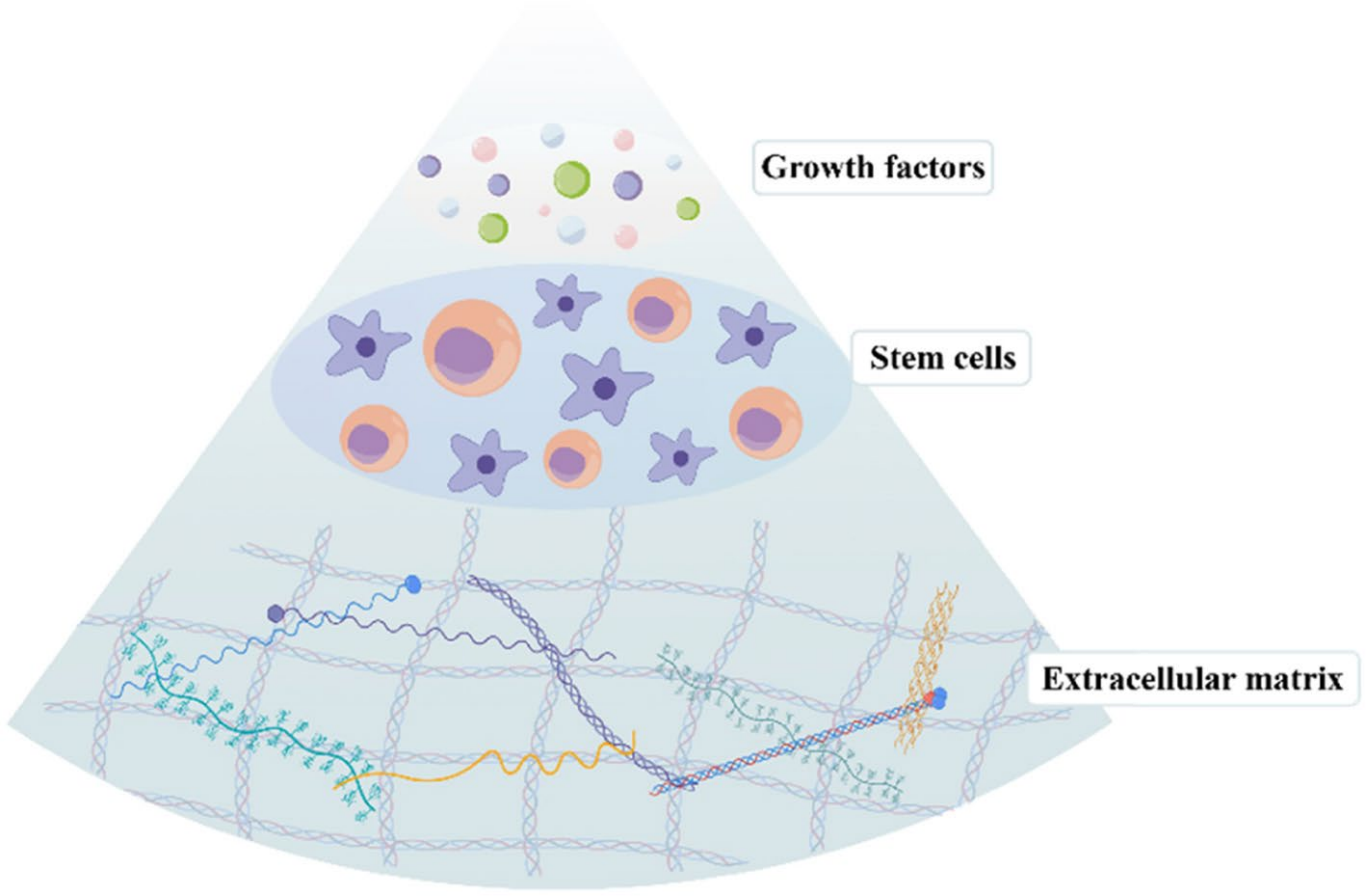
Clinically valuable components of AM. The therapeutic potential of AM is attributed to its key biological components: the extracellular matrix, resident stem cells, and a repertoire of growth factors

Human amniotic membrane (hAM) represents a multifunctional biomaterial harbouring growth factors, stem cells and extracellular matrix, with considerable potential in tissue repair and regenerative medicine (Fig. 2). These effects are mediated through the secretion of bioactive factors by human amniotic epithelial cells (hAECs) and human amniotic mesenchymal stromal cells (hAMSCs), including interleukin-1 receptor antagonist (IL-1Ra) and tissue inhibitors of metalloproteinases (TIMP-1, -2, -3, -4) (Jafari et al. 2024). These factors suppress fibroblast activation, thereby reducing tissue adhesion and scar formation. The low immunogenicity of hAM is primarily mediated by the expression of immunomodulatory molecules, including human leukocyte antigen‑G (HLA‑G) and factor-related apoptosis ligand (FasL) (Strom and Gramignoli 2016). In addition, hAECs and hAMSCs secrete multiple growth factors such as bFGF and HGF, which collectively promote cell proliferation, migration, and tissue regeneration (Table 1). Structurally, the basement membrane of AM is rich in collagen and fibronectin, while its avascular and densely organized structure provides favourable mechanical support and a pro-regenerative milieu for cellular adhesion and tissue repair (Fenelon et al. 2021).

**Table 1:** Profile of growth factors in AM: Categories, functions, and release concentrations. (Measured from the amniotic stroma within 24 h at 37 °C)

Growth Factor	Abbreviation	Primary Function	Concentration (pg/cm^2^)
Tissue Inhibitor of Metalloprotease-2	TIMP-2	Regulation of extracellular matrix degradation and remodelling	1911
Hepatocyte Growth Factor	HGF	Promotion of proliferation, differentiation, and migration in epithelial and endothelial cells	1213
Transforming Growth Factor β1	TGF-β1	Regulation of cell proliferation and differentiation, with anti-inflammatory and immunomodulatory effects	332
Tissue Inhibitor of Metalloprotease-3	TIMP-3	Inhibition of extracellular matrix degradation	251
Insulin-like Growth Factor 1	IGF-1	Promotion of growth and proliferation in cartilage and endothelial cells	98
Platelet-Derived Growth Factor BB	PDGF-BB	Promotion of smooth muscle, endothelial, and cartilage cell proliferation, wound healing, and angiogenesis	97
Insulin-like Growth Factor 2	IGF-2	Promotion of cell growth and proliferation	90
Growth-Related Oncogene α	GRO-α	Promotion of leukocyte migration and inflammatory responses	60
Transforming Growth Factor β2	TGF-β2	Involvement in immunosuppression, anti-inflammatory effects, and tissue repair	54
Tissue Inhibitor of Metalloprotease-4	TIMP-4	Maintenance of extracellular matrix homeostasis	53
Interleukin-1 Receptor Antagonist	IL-1Ra	Inhibition of inflammatory responses	49
Epidermal Growth Factor	EGF	Promotion of epidermal cell proliferation and wound healing	45
Basic Fibroblast Growth Factor	bFGF	Stimulation of angiogenesis and cell proliferation	32
Monocyte Chemoattractant Protein-1	MCP-1	Chemoattraction of monocytes to inflammatory sites	26
Interleukin-6	IL-6	Modulation of inflammatory responses and immune cell communication	15
Transforming Growth Factor α	TGF-α	Stimulation of cell proliferation, particularly in epidermal cells	12
Tumour Necrosis Factor α	TNF-α	Involvement in inflammatory and immune responses	1

The concentration data were obtained from the product manual of Arthrex Amnion™ Matrix

**Fig. 3:**
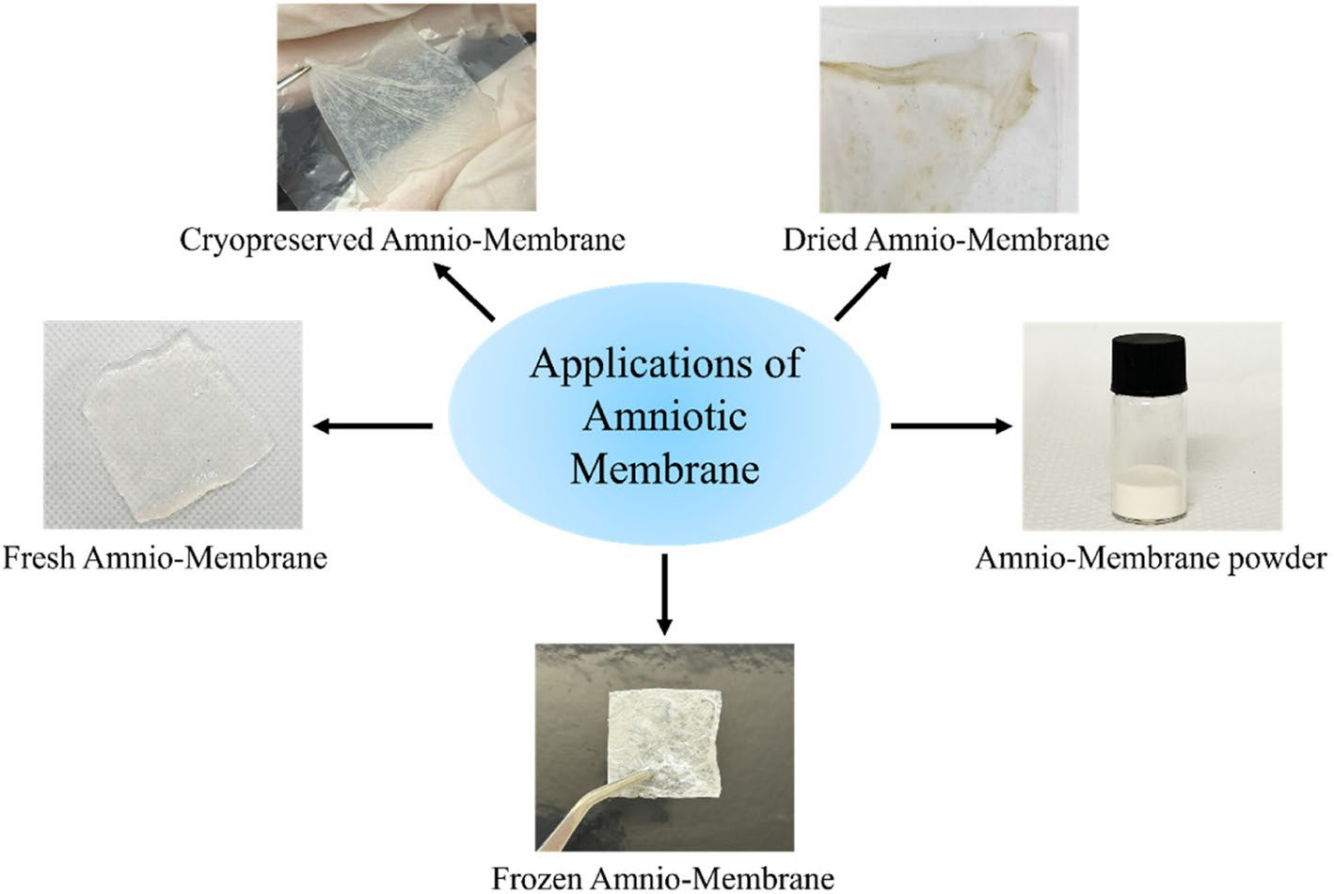
Schematic representation of the different clinical application forms of processed AM

Currently, AM is clinically utilized primarily in several processed forms, including cryopreserved, dehydrated, and powdered AM (Fig. 3). These formulations have been widely adopted due to their ease of storage and handling while retaining key biological functions. This review systematically summarizes recent advances in the clinical application of AM across multiple disciplines, including orthopaedics (e.g., tendon repair, osteoarthritis management, and prevention of postoperative adhesion), obstetrics and gynaecology (e.g. repair of endometrial injury), and dermatology (e.g. treatment of diabetic foot ulcer and enhancement of burn wound healing). In addition, the underlying biological mechanisms that mediate these therapeutic effects are discussed.

## Applications of AM in orthopaedics

Musculoskeletal disorders are among the leading causes of disability worldwide, accounting for approximately 17% of the total burden (Cieza et al. 2021). These conditions substantially impair mobility and quality of life. With the ongoing trend of population aging, the incidence of musculoskeletal disorders continues to rise, underscoring an urgent need for effective tissue repair strategies and advanced biomaterials (Gonzalez-Fernandez et al. 2019).

### Application in tendon repair

Currently available tendon repair materials—including synthetic polymer scaffolds (e.g., PCL, PLA), natural polymer scaffolds (e.g., collagen, chitosan), and decellularized extracellular matrix (dECM) scaffolds—each exhibit their own inherent limitations. Synthetic scaffolds often suffer from low porosity, which restricts cellular infiltration and compromises biological activity. Natural scaffolds frequently lack sufficient mechanical strength, while dECM scaffolds are limited by source scarcity and batch-to-batch variability (Hou et al. 2016).

In contrast, hAM possesses distinct advantages for tendon repair due to its unique composition and structure. Rich in collagen, fibronectin, and a wide range of bioactive growth factors, hAM creates a pro-regenerative microenvironment that supports the adhesion, proliferation, and differentiation of tendon stem/progenitor cells (Fenelon et al. 2021). Furthermore, its mechanical properties can be enhanced through chemical crosslinking using agents such as polyethylene glycol dialdehyde (PEG-dialdehyde) (Skopinska-Wisniewska et al. 2023).

Beyond structural and biochemical advantages, hAM exerts strong immunomodulatory and anti-inflammatory effects. Key mediators, including IL‑10 and thrombospondin 1 (TSP‑1), regulate the local repair milieu by promoting macrophage polarization toward a pro-healing phenotype, enhancing angiogenesis, and inhibiting matrix metalloproteinase (MMP) activity (Moreno et al. 2022; Zhang et al. 2024b).

Notably, hAM effectively prevents postoperative adhesions. For example, a clinical study reported that over 90% of patients treated with micronized dehydrated hAM injections exhibited significant pain relief and reduced adhesion-related symptoms within three months (Gellhorn and Han 2017).

### Amelioration of osteoarthritis

Osteoarthritis is a highly prevalent degenerative joint disease with an increasing global incidence (Wu et al. 2025). Current intra-articular therapies include pharmaceuticals (e.g., corticosteroids), biologics (e.g., platelet-rich plasma, stem cells), and biomedical materials (e.g., hyaluronic acid, chitosan). Despite widespread use, their long-term disease-modifying effects remain limited. Recently, injectable AM powder has emerged as a promising alternative for osteoarthritis management.

The therapeutic value of AM stems from its diverse bioactive properties. First, its high content of anti-inflammatory mediators, such as TGF-β and IL‑10, effectively suppresses pro-inflammatory cytokines in the osteoarthritic joint, alleviating synovitis and slowing cartilage degradation (Askari et al. 2026). Preclinical studies have demonstrated that intra‑articular AM administration significantly reduces IL‑1β and tumour necrosis factor-α (TNF-α) levels while increasing IL-10 and IL-1Ra expression, thereby effectively reversing the pro-inflammatory joint microenvironment (Bhattacharjee et al. 2022).

In addition to immunomodulation, AM supports cartilage preservation and repair. Specifically, AM upregulates TIMP expression, inhibiting MMP-mediated extracellular matrix degradation. This TIMP-dependent mechanism helps maintain cartilage structural integrity and creates a favourable microenvironment for chondrocyte survival, proliferation, and matrix synthesis (Fenelon et al. 2021).

### Prevention of epidural fibrosis after spinal surgery

Due to its intrinsic anti‑inflammatory, anti‑fibrotic, and physical barrier characteristics (Fenelon et al. 2021), AM has been widely explored for reducing epidural fibrosis and scar formation following spinal surgery.

In a canine multi-level laminectomy model, glutaraldehyde-crosslinked AM applied over the dura mater effectively preserved the epidural space. At 12 weeks postoperatively, only sparse, loose connective tissue was observed, in contrast to the dense fibrotic adhesions in the control group. Histological analyses further revealed a 51% reduction in fibroblast infiltration, significantly decreased collagen deposition, and a markedly attenuated inflammatory response (Tao and Fan 2009), indicating strong inhibition of fibrotic progression.

Comparative studies have also highlighted the importance of AM processing methods for anti-adhesion efficacy. Freeze‑dried AM resorbs completely within 6 weeks, providing only temporary barrier protection. In contrast, crosslinked AM degrades more slowly and maintains structural integrity longer. Notably, its anti‑adhesion performance is comparable to autologous free fat grafts, but with superior cost-effectiveness and no donor-site morbidity (Tao and Fan 2009). These advantages make crosslinked AM particularly suitable for long-segment spinal procedures or patients with insufficient autologous adipose tissue.

In summary, crosslinked hAM represents a safe, low-immunogenicity, and effective biological barrier for preventing postoperative epidural fibrosis.

## Applications of AM in obstetrics and gynaecology

Global health surveys indicate that by 2010, an estimated 48.5 million couples worldwide were affected by infertility, with endometrial injury being a predominant cause of female infertility (Mascarenhas et al. 2012). Damage to the endometrium basal layer—resulting from intrauterine surgical procedures, infection, or other pathological insults—frequently leads to intrauterine adhesions, endometrial thinning, and compromised endometrial receptivity. Current therapeutic approaches, including hormonal therapy and surgical adhesiolysis, show limited efficacy and are associated with high recurrence rates (Liu et al. 2023). Accordingly, there is an urgent demand for regenerative strategies that can restore both the structural integrity and functional competence of the endometrium. In this setting, hAM has emerged as a promising candidate due to its inherent pro-regenerative biological properties.

Unlike conventional materials such as collagen sponges or hyaluronic acid gels, which primarily provide passive physical support, AM exhibits superior bioactivity and the capacity to actively promote tissue regeneration (Li et al. 2019a). As a bioactive scaffold, AM offers a three‑dimensional framework that supports cell adhesion, migration, and proliferation, while also acting as a physical barrier to reduce postoperative adhesion formation (Heidari et al. 2025). Furthermore, the epithelial and stromal layer of AM is enriched with a diverse array of growth factors and anti‑inflammatory mediators, including epidermal growth factor (EGF), vascular endothelial growth factor (VEGF), TGF‑β, and IL‑1Ra (Jafari et al. 2024; Li et al. 2021). These bioactive factors act in concert to boost endometrial cell proliferation and angiogenesis, while modulating the local immune microenvironment to restrain excessive inflammation and fibrotic reactions.

Preclinical data further corroborate the regenerative potential of hAM. In a rat model of endometrial injury, transplantation of hAM-derived hAMSCs led to significant increases in endometrial thickness, glandular density, and vascularization. These structural benefits were accompanied by reduced expression of the pro‑inflammatory cytokine TNF‑α and upregulated expression of collagen type I alpha 1 chain (COL1A1), collectively indicating improved endometrial regeneration and enhanced uterine receptivity (Gan et al. 2017).

Taken together, these findings highlight the notable potential of hAM to optimize reproductive outcomes in patients receiving assisted reproductive technologies. While definitive clinical validation requires further investigation in large-scale, prospective, randomized controlled trials, existing evidence strongly supports hAM as an innovative and promising regenerative tool in obstetrics and gynaecology.

## Applications of AM in dermatology

Chronic non-healing wounds, such as diabetic foot ulcer (DFU) and severe burn injury, pose a significant and escalating global healthcare burden. DFU, which primarily results from peripheral neuropathy and vascular dysfunction, is characterized by delayed wound healing, high susceptibility to infection, and extensive soft tissue damage (Burgess et al. 2021). In contrast, severe burns are typically associated with pronounced inflammatory response, significantly impaired regenerative capacity, and a high risk of hypertrophic scarring and contracture (Radzikowska-Büchner et al. 2023). Conventional inert wound dressings (e.g., gauze, films) serve merely as physical barriers to retain moisture and cover wounds, without actively promoting the healing process. Although advanced biomaterials such as collagen sponges and synthetic hydrogels have been developed, their capacity to dynamically modulate the pathological wound microenvironment remains suboptimal. In this context, hAM has gained increasing interest as a natural biomaterial with excellent biocompatibility and multifunctional reparative properties, which are particularly advantageous for chronic wound management.

The therapeutic efficacy of hAM stems from its multifaceted biological mechanisms. Specifically, it accelerates re-epithelialization and promotes neurovascular regeneration through the sustained release of numerous bioactive factors. In addition, hAM exhibits intrinsic antimicrobial activity and functions as a protective and analgesic barrier, thereby reducing bacterial colonization and alleviating wound-associated pain (Abdullah Alghamdi et al. 2024). Collectively, these properties enable hAM to actively regulate wound healing by remodelling the pathological microenvironment and facilitating ordered tissue repair.

### Treatment of DFU

DFU, a severe diabetic complication, faces multiple pathophysiological healing barriers, including persistent hyperglycaemia, chronic inflammation, neurovascular dysfunction, and recurrent infection. These interconnected factors contribute to the limited efficacy of conventional therapies and high ulcer recurrence rates (Bandyk 2018; Huang et al. 2023). In this context, hAM has emerged as a promising therapeutic strategy for DFU management. The reparative effects of hAM are primarily mediated by its modulation of the inflammatory wound microenvironment. For instance, in combination with mesenchymal stem cells, hAM significantly downregulates the expression of pro-inflammatory factors such as TNF-α and IL-1β, thereby attenuating chronic inflammation and facilitating tissue repair (Abdullah Alghamdi et al. 2024). Additionally, hAM functions as a three-dimensional scaffold that supports cell migration and angiogenesis, thereby promoting granulation tissue formation and re-epithelialization. Experimental studies have demonstrated that stem cell-loaded hAM scaffolds significantly accelerate the healing of chronic diabetic wounds (Hashemi et al. 2019; Hashemi et al. 2021). Beyond its regenerative capacity, hAM exhibits intrinsic analgesic and antibacterial properties. Specifically, the extracellular matrix component heavy chain-hyaluronic acid/pentraxin-3 (HC-HA/PTX3) complex suppresses nociceptive neuronal excitation via a CD44 receptor-dependent pathway. This mechanism blocks pain signalling effectively without the adverse effects of opioid analgesics (Zhang et al. 2024a).

Unlike single-agent hydrogel delivery systems, hAM is a naturally integrated bioactive composite scaffold enriched with multiple growth factors (EGF, VEGF, FGF). Thus, it serves not only as a delivery platform but also autonomously establishes a pro-regenerative microenvironment that supports sustained wound healing (Bardill et al. 2022; Li et al. 2023). Clinical evidence further supports its therapeutic potential: randomized controlled trials have shown that dehydrated hAM products, when used as an adjunct to standard wound care, significantly improve complete ulcer closure rates and reduce healing time compared to standard care alone (Mohammed et al. 2022).

### Promotion of burn wound healing

The management of severe burns aims to achieve rapid wound coverage, infection control, dermal regeneration, and the prevention of excessive scar formation. Although autologous skin grafting remains the gold standard, its application is often limited by donor-site morbidity and restricted availability. As an alternative biological dressing, hAM exerts multiple therapeutic mechanisms that synergistically facilitate burn wound repair (Guo et al. 2024). By providing a moist physical barrier and delivering a broad spectrum of bioactive molecules, hAM actively promotes epithelial cell proliferation and migration. Notably, factors secreted by AM-derived mesenchymal stem cells, including plasminogen activator inhibitor-1 (PAI-1), granulocyte colony-stimulating factor (G-CSF), and TIMP-1, suppress heat-induced apoptosis in skin cells, thereby accelerating wound healing. This protective effect is primarily mediated by the activation of pro-survival signalling pathways, particularly the phosphatidylinositol-3-kinase/protein kinase B (PI3K/AKT) pathway (Li et al. 2019b). Owing to its natural origin, hAM demonstrates superior biocompatibility and lower inflammatory reactivity than many synthetic wound dressings (Naasani et al. 2022). Clinical evidence further substantiates the therapeutic value of hAM in burn care. For instance, in paediatric burn patients, dehydrated hAM yields wound healing outcomes equivalent to split-thickness skin grafting, while significantly reducing hypertrophic scarring and contracture (Ahuja et al. 2020). Moreover, the use of hAM as a scaffold for mesenchymal stem cell delivery has shown promising results in preclinical models, enhancing tissue regeneration quality and modulating the inflammatory response (Naasani et al. 2022).

To address the intrinsic limitations of hAM, such as insufficient mechanical strength and rapid in vivo degradation, various modification strategies have been explored, including chemical cross-linking and composite fabrication. For example, glutaraldehyde cross-linking has been demonstrated to notably enhance the mechanical stability and degradation resistance of hAM (Bhawna et al. 2023). Additionally, integration with platelet-rich plasma further increases local growth factor availability and improves wound adherence, resulting in accelerated healing and reduced scar formation in an animal model (Kshersagar et al. 2018). Collectively, these modification approaches significantly broaden the clinical applicability of hAM in burn wound management.

## Conclusions and perspectives

This review systematically summarizes recent advances and therapeutic potential of the hAM as a versatile natural biomaterial across diverse disciplines, including orthopaedics, obstetrics and gynaecology, and dermatology. Its wide-ranging applications—from tendon repair and osteoarthritis management to endometrial regeneration, diabetic foot ulcer healing, and burn wound treatment—reflect a key paradigm shift in regenerative medicine. HAM has evolved from a passive biological dressing into an active regulator of the tissue repair microenvironment.

Currently, hAM-based formulations have shown promising outcomes in both preclinical investigations and clinical settings. Nevertheless, several critical directions should be prioritized to fully exploit their translational potential: (1) Improving the mechanical properties of hAM is essential for repairing large tissue defects or for use in load-bearing environments. Strategies including chemical cross-linking and composite preparation can enhance its mechanical strength and stability, thereby broadening its applicability to load-bearing orthopaedic repairs and gynaecological procedures that require structural support. (2) Optimizing preservation methods. Most commercial hAM products employ lyophilization or hydrated cryopreservation. Lyophilized membranes tend to be brittle, whereas cryopreserved products depend on strict cold-chain logistics. The development of alternative methods, such as glycerination, which maintains structural integrity and bioactivity while simplifying storage, would significantly improve clinical availability and application.

Looking forward, continuous advances in regenerative medicine and biomaterials engineering will enable precise modulation of hAM’s structure and bioactivity. These advances are expected to not only strengthen its therapeutic efficacy in established indications but also extend its scope to the regeneration of more complex tissues, including those of the nervous and urogenital systems.

## Data Availability

Not applicable.

## Abbreviations

HAM/hAM: Human amniotic membrane
IL-1Ra: Interleukin-1 receptor antagonist
TIMP: Tissue inhibitor of metalloproteinase
hAECs: Human amniotic epithelial cells
hAMSCs: Human amniotic mesenchymal stem cells
HLA-G: Human leukocyte antigen-G
FasL: Factor-related apoptosis ligand
PCL: Polycaprolactone
PLA: Polylactic acid
TGF: Transforming growth factor
dECM: Decellularized extracellular matrix
PEG-dialdehyde: Polyethylene glycol dialdehyde
TSP-1: Thrombospondin-1
MMP: Matrix metalloproteinase
TNF-α: Tumour necrosis factor α
EGF: Epidermal growth factor
VEGF: Vascular endothelial growth factor
COL1A1: Collagen type I alpha 1 chain
DFU: Diabetic foot ulcer
HC-HA/PTX3: Heavy chain-hyaluronic acid/pentraxin 3
FGF: Fibroblast growth factor
PAI-1: Plasminogen activator inhibitor-1
G-CSF: Granulocyte colony-stimulating factor
PI3K/AKT: Phosphatidylinositol-3-kinase/protein kinase B
